# Using WeChat, a Chinese Social Media App, for Early Detection of the COVID-19 Outbreak in December 2019: Retrospective Study

**DOI:** 10.2196/19589

**Published:** 2020-10-05

**Authors:** Wenjun Wang, Yikai Wang, Xin Zhang, Xiaoli Jia, Yaping Li, Shuangsuo Dang

**Affiliations:** 1 Second Affiliated Hospital of Xi'an Jiaotong University Xi'an China

**Keywords:** novel coronavirus, SARS, SARS-CoV-2, COVID-19, social media, WeChat, early detection, surveillance, infodemiology, infoveillance

## Abstract

**Background:**

A novel coronavirus, SARS-CoV-2, was identified in December 2019, when the first cases were reported in Wuhan, China. The once-localized outbreak has since been declared a pandemic. As of April 24, 2020, there have been 2.7 million confirmed cases and nearly 200,000 deaths. Early warning systems using new technologies should be established to prevent or mitigate such events in the future.

**Objective:**

This study aimed to explore the possibility of detecting the SARS-CoV-2 outbreak in 2019 using social media.

**Methods:**

WeChat Index is a data service that shows how frequently a specific keyword appears in posts, subscriptions, and search over the last 90 days on WeChat, the most popular Chinese social media app. We plotted daily WeChat Index results for keywords related to SARS-CoV-2 from November 17, 2019, to February 14, 2020.

**Results:**

WeChat Index hits for “Feidian” (which means severe acute respiratory syndrome in Chinese) stayed at low levels until 16 days ahead of the local authority’s outbreak announcement on December 31, 2019, when the index increased significantly. The WeChat Index values persisted at relatively high levels from December 15 to 29, 2019, and rose rapidly on December 30, 2019, the day before the announcement. The WeChat Index hits also spiked for the keywords “SARS,” “coronavirus,” “novel coronavirus,” “shortness of breath,” “dyspnea,” and “diarrhea,” but these terms were not as meaningful for the early detection of the outbreak as the term “Feidian”.

**Conclusions:**

By using retrospective infoveillance data from the WeChat Index, the SARS-CoV-2 outbreak in December 2019 could have been detected about two weeks before the outbreak announcement. WeChat may offer a new approach for the early detection of disease outbreaks.

## Introduction

An outbreak of pneumonia of unknown cause in Wuhan, the capital of Hubei province, China, occurred in December 2019 [1]. Shortly, the cause was identified as a novel coronavirus [1] that resembles severe acute respiratory syndrome (SARS) and it was named SARS-CoV-2 [2,3]. The outbreak has become a pandemic, with 2.7 million confirmed cases and nearly 200,000 deaths globally as of April 24, 2020 [4]. Early warning systems should be established to prevent or mitigate future disease outbreaks.

Traditional surveillance systems typically rely on clinical, virological, and microbiological data submitted by physicians and laboratories. Due to time and resource constraints, a lack of operational knowledge of reporting systems, and regulations associated with these systems, substantial lags between an outbreak event and its report are common [5].

With the popularization of the internet and smartphones, an increasing number of people use social media (eg, Twitter and Facebook) to share information. Details of an event may have been posted about on social media for several days or even months before it was reported through health institutions and official reporting structures. Internet-based search engines are an important source for health information for people from all walks of life. Analyzing data on search behaviors provides a new approach for the detection and monitoring of diseases and symptoms. Technologies using social media, search queries, and other internet resources offer novel and economic approaches for detecting and tracking emerging diseases and such approaches (called infodemiology and infoveillance) have been successfully used in the cases of SARS [6], influenza [7], and dengue [8]. Herein, we explored whether the SARS-CoV-2 outbreak in China could have been detected earlier through data available on WeChat, a popular Chinese social media app. Internet search queries from Hubei province were also investigated.

## Methods

WeChat (called Weixin in China; Tencent Inc) is the most popular social media app in China with over 1 billion monthly active users. WeChat Index, accessed on the WeChat app, is a publicly available data service that shows how frequently a specific keyword has appeared in posts, subscriptions, and search on WeChat over the previous 90 days. Using WeChat Index, we obtained daily data from November 17, 2019, to February 14, 2020, for keywords related to SARS-CoV-2, such as “SARS,” “Feidian” (SARS in Chinese), “pneumonia,” “fever,” “cough,” “shortness of breath,” “dyspnea,” “fatigue,” “stuffy nose,” “runny nose,” “diarrhea,” “coronavirus,” “novel coronavirus,” and “infection” (raw data in Multimedia Appendix 1). The corresponding Chinese words were used for all keywords except for “SARS”.

Baidu is the dominant Chinese internet search engine. Baidu Index (Baidu Inc) [9] can display how frequently a keyword has been queried over a certain time period in a given region. The keywords mentioned above were also investigated through Baidu Index for Hubei province.

The daily data were plotted according to time for each of the keywords. As the outbreak is an isolated rather than recurrent event and the cutoff value to detect an outbreak based on social media and online search behavior is unknown, statistical analyses were not performed. The outbreak was announced by Wuhan Health Commission (WHC) on December 31, 2019; on this day, the Chinese Centers for Disease Control and Prevention (China CDC) became involved in the investigation and response [2]. If WeChat Index results for a keyword spiked or increased before the day of the outbreak announcement, the keyword was considered as a potential candidate outbreak sign [10].

## Results

**Figure 1:**
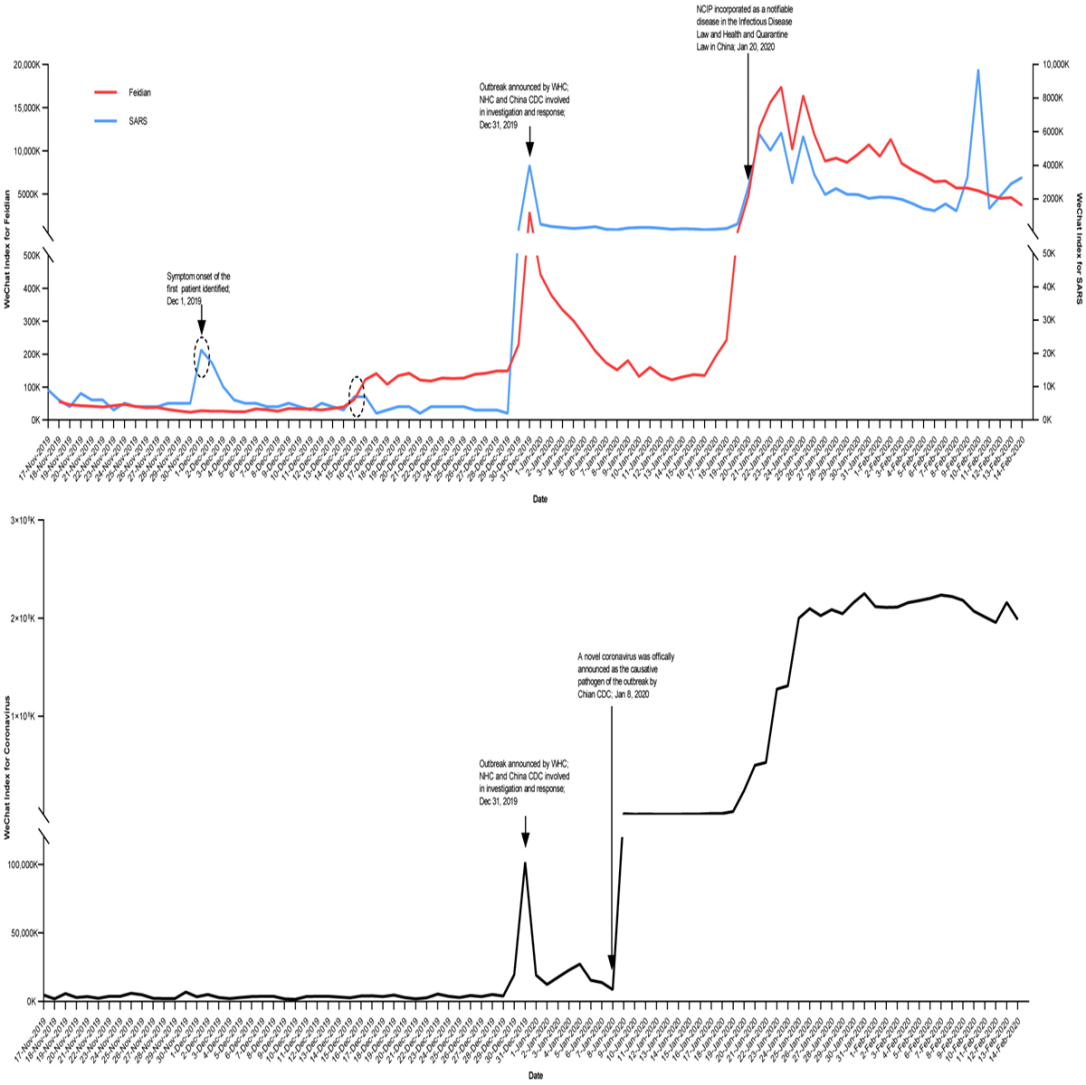
WeChat Index results for the words Feidian, SARS, and coronavirus. The index results for “Feidian” began to rise on December 15, 2019 (dashed circle), persisted at relatively high levels until December 29, 2019, and rose rapidly on December 30, 2019, with a peak on December 31, 2019. The index results for “SARS” were atypical during the first three days of December, with a peak on December 1, 2019 (dashed circle). The index results for “coronavirus” began to rise on December 30, 2019, with a peak on December 31, 2019, followed by another increase on January 9, 2020. China CDC: Chinese Centers for Disease Control and Prevention; Feidian: Chinese abbreviation of severe acute respiratory syndrome; NCIP: novel coronavirus-infected pneumonia; NHC: National Health Commission of the People's Republic of China; SARS: severe acute respiratory syndrome.

WeChat Index hits for “Feidian” stayed at low levels before December 15, 2019, after which they increased significantly. The WeChat Index results remained at relatively high levels until the day before the outbreak announcement, when the number of hits rose rapidly, reaching a peak on the day of the outbreak announcement (Figure 1). The WeChat Index results for “SARS” were stable, except for the first three days in December, with a peak on December 1, 2019 (Figure 1). The WeChat Index hits for “coronavirus” rose the day before the outbreak was announced, with a peak on the day of the announcement, followed by another peak after the novel coronavirus was officially announced as the causative pathogen of the outbreak by China CDC (Figure 1). From November 17, 2019, to December 30, 2019 (44 days), the WeChat Index results also spiked or increased for “novel coronavirus,” “shortness of breath,” “dyspnea,” and “diarrhea,” although these terms were not as meaningful for the early detection of the outbreak as “Feidian” (Multimedia Appendices 2 and 3).

The Baidu Index results for “Feidian,” “SARS,” “pneumonia,” and “coronavirus” rose rapidly on December 30, 2019, the day before the outbreak announcement. According to Baidu Index results, no other keywords had an obvious increase from November 17, 2019, to December 30, 2019 (Multimedia Appendix 4).

## Discussion

### Principal Results

By exploring daily data from WeChat, a Chinese social media app, we found that the posting and search frequencies of several keywords related to SARS-CoV-2 deviated from typical frequencies ahead of the outbreak being announced in China in December 2019. Of these keywords, “Feidian” is especially worthy of attention. In 2003, the SARS outbreak caused mass panic among people in China and approximately half of the victims were health care workers [11]. Since then, Chinese physicians are on the alert for SARS as well as similar diseases [12]. If the clinical manifestations and chest images indicate viral pneumonia and several similar cases occur in a region in a short period, health care providers may think of SARS (“Feidian” in Chinese). When suspected cases are admitted to hospitals, the involved physicians may mention “Feidian” and communicate on WeChat using this word. This study found that the frequency of the word “Feidian” in WeChat began to rise on December 15, 2019. According to publications regarding early cases of laboratory-confirmed SARS-CoV-2 infections, 5-11 patients had symptom onset by this day; the earliest onset was on December 1, 2019 [1,2]. Furthermore, the WeChat Index results for “Feidian” persisted at levels higher than those prior to December 15, 2019, and they reached a peak the day of the outbreak announcement. Altogether, the WeChat Index results for the word “Feidian” offered a strong warning sign of the developing SARS-CoV-2 outbreak. Using WeChat data in this way may enable the early detection of future outbreaks; for SARS-CoV-2, this data indicated an outbreak two weeks before the outbreak announcement.

The frequency of the term “SARS” in WeChat was unusually high from December 1 to 3, 2019, compared to the days before and after. According to Huang et al [13], the symptom onset date of the first patient identified was December 1, 2019. It is not clear whether this frequency abnormality is related to early cases. If it is, it indicates the existence of cases prior to the first reported one. The frequency of “novel coronavirus” in WeChat was abnormally high on December 11, 2019, with an index value of 400. However, its baseline value (0 or 50) was very low, so the index was sensitive to noise (Multimedia Appendix 3). The frequency of the word “coronavirus” in WeChat rose rapidly one day ahead of the outbreak announcement, so the role of this keyword was limited in the early detection of this outbreak. As for keywords related to symptoms, these symptoms are not specific to SARS-CoV-2 infection. Their increased frequency may be associated with the emergence of COVID-19, or it may be a coincidence. Although the other keywords explored in this study did not perform as well as “Feidian,” both these terms and keywords not explored in this study (eg, the names of drugs used to treat SARS-CoV-2 infection) may still prove valuable for future outbreak detection and monitoring. A previous investigation using Google Flu Trends showed that a combination of several keywords was better than a single keyword for making predictions [7].

"Infoveillance", which is the gathering and analyzing data from social media, internet search queries, and information from websites for infodemiology purposes, was proposed in 2004 by Eysenbach as a novel approach to early warning and detection of either disease outbreaks or infodemics. Infoveillance can be supplementary to traditional surveillance systems [5]. One such tool, the Global Public Health Intelligence Network (GPHIN), identified the SARS outbreak in China in 2003 more than two months earlier. In addition, they identified the outbreak of Middle East respiratory syndrome (MERS) in 2012 [6]. As far as we know, GPHIN and other established tools do not gather data from WeChat, the dominant Chinese social media app. This study shows that gathering and analyzing data from WeChat may be promising for the early detection of disease outbreaks. Considering WeChat has over 1 billion monthly active users in China, it has an advantage in detecting outbreaks within China. In addition, we found that WeChat data may provide better results than Baidu search query data because people may primarily communicate with others using WeChat [14].

### Limitations

The main limitation of this study is its retrospective nature. The outbreak is a singular event. Using WeChat data for the early detection of outbreaks like this one should be further explored in the future. In addition, WeChat Index data earlier than 90 days ago is unavailable and the index calculation methodology is not public.

### Conclusions

In summary, data from WeChat could have enabled the detection of the SARS-CoV-2 outbreak in 2019 about two weeks earlier than the outbreak announcement. Future studies can prospectively gather and analyze data from WeChat for the early detection of disease outbreaks in China. Tracking the source of keywords in WeChat that have atypical frequencies may become a promising approach for controlling a disease outbreak at its earliest stages.

## Appendix

Multimedia Appendix 1 Raw data of WeChat Index for keywords related to SARS-CoV-2.

Multimedia Appendix 2 Keywords for which WeChat Index spiked or increased during the period from November 17 to December 30, 2019.

Multimedia Appendix 3 WeChat Index curves for keywords related to SARS-CoV-2, other than “Feidian” and “SARS”.

Multimedia Appendix 4 Baidu Index curves for keywords related to SARS-CoV-2.

## Abbreviations

China CDC: Chinese Centers for Disease Control and Prevention
GPHIN: Global Public Health Intelligence Network
MERS: Middle East respiratory syndrome
SARS: severe acute respiratory syndrome
WHC: Wuhan Health Commission

## References

[1] Lu R, Zhao X, Li J, Niu P, Yang B, Wu H, Wang W, Song H, Huang B, Zhu N, Bi Y, Ma X, Zhan F, Wang L, Hu T, Zhou H, Hu Z, Zhou W, Zhao L, Chen J, Meng Y, Wang J, Lin Y, Yuan J, Xie Z, Ma J, Liu WJ, Wang D, Xu W, Holmes EC, Gao GF, Wu G, Chen W, Shi W, Tan W (2020). Genomic characterisation and epidemiology of 2019 novel coronavirus: implications for virus origins and receptor binding. Lancet.

[2] Zhu N, Zhang D, Wang W, Li X, Yang B, Song J, Zhao X, Huang B, Shi W, Lu R, Niu P, Zhan F, Ma X, Wang D, Xu W, Wu G, Gao GF, Tan W (2020). A Novel Coronavirus from Patients with Pneumonia in China, 2019. N Engl J Med.

[3] Gorbalenya AE, Baker SC, Baric RS (2020). Severe acute respiratory syndrome-related coronavirus: The species and its viruses - a statement of the Coronavirus Study Group. BioRxiv.

[4] Coronavirus disease (COVID-2019) situation reports. World Health Orgnization.

[5] Milinovich GJ, Williams GM, Clements ACA, Hu W (2014). Internet-based surveillance systems for monitoring emerging infectious diseases. The Lancet Infectious Diseases.

[6] Dion M, AbdelMalik P, Mawudeku A (2015). Big Data and the Global Public Health Intelligence Network (GPHIN). Can Commun Dis Rep.

[7] Ginsberg J, Mohebbi MH, Patel RS, Brammer L, Smolinski MS, Brilliant L (2009). Detecting influenza epidemics using search engine query data. Nature.

[8] Chan EH, Sahai V, Conrad C, Brownstein JS (2011). Using web search query data to monitor dengue epidemics: a new model for neglected tropical disease surveillance. PLoS Negl Trop Dis.

[9] Baidu Index.

[10] Mohsin M, Hamdan A, Bakar A (2012). Review on anomaly detection for outbreak detection.

[11] Wenzel RP, Bearman G, Edmond MB (2005). Lessons from severe acute respiratory syndrome (SARS): implications for infection control. Arch Med Res.

[12] Zhong NS, Zeng GQ (2008). Pandemic planning in China: applying lessons from severe acute respiratory syndrome. Respirology.

[13] Huang C, Wang Y, Li X, Ren L, Zhao J, Hu Y, Zhang L, Fan G, Xu J, Gu X, Cheng Z, Yu T, Xia J, Wei Y, Wu W, Xie X, Yin W, Li H, Liu M, Xiao Y, Gao H, Guo L, Xie J, Wang G, Jiang R, Gao Z, Jin Q, Wang J, Cao B (2020). Clinical features of patients infected with 2019 novel coronavirus in Wuhan, China. The Lancet.

[14] Tu F (2016). WeChat and civil society in China. Communication and the Public.

