# Supplementary material for: Using WeChat, a Chinese Social Media App, for Early Detection of the COVID-19 Outbreak in December 2019: Retrospective Study
Source: JMIR Mhealth Uhealth. 2020 Oct 5;8(10):e19589. doi: 10.2196/19589 (PMC7572119; doi:10.2196/19589)
Supplement: Multimedia Appendix 2 [file mhealth_v8i10e19589_app2.docx]

**Supplementary Table: Keywords for which WeChat Index spiked or increased during the period from Nov 17, 2019 to Dec 30, 2019.**

| Keyword | A-day | The time from A-day to D-day |
| --- | --- | --- |
| Feidian | Dec 15, 2019 | 16 days |
| SARS | Dec 01, 2019 | 30 days |
| Coronavirus | Dec 30, 2019 | 1 day |
| Novel coronavirus | Dec 11, 2019 | 20 days |
| Shortness of breath | Dec 22, 2019 | 9 days |
| Dyspnea | Dec 22, 2019 | 9 days |
| Diarrhea | Dec 18, 2019 | 13 days |

A-day: the day when WeChat index for a keyword spiked or began to increase during the period from Nov 17, 2019 to Dec 30, 2019.

D-day: Dec 31, 2019, the day that the SARS-Cov-2 outbreak was announced by Wuhan Health Commission and Chinese Center for Disease Control and Prevention involved in investigation and response.

WeChat Index for novel coronavirus had a spike on Dec 11, 2019. WeChat Index for coronavirus had been roughly stable till Dec 30, 2019, when it rose rapidly WeChat Index for shortness of breath as well as dyspnea began to increase from Dec 22, 2019. WeChat Index for diarrhea spiked on Dec 18, 2019. WeChat Index for pneumonia had spikes but showed a periodic pattern with a 7-day cycle without obvious long-term trend so that was not considered as a potential candidate for the outbreak sign.
