# Supplementary material for: Using WeChat, a Chinese Social Media App, for Early Detection of the COVID-19 Outbreak in December 2019: Retrospective Study
Source: JMIR Mhealth Uhealth. 2020 Oct 5;8(10):e19589. doi: 10.2196/19589 (PMC7572119; doi:10.2196/19589)
Supplement: Multimedia Appendix 3 [file mhealth_v8i10e19589_app3.pdf]

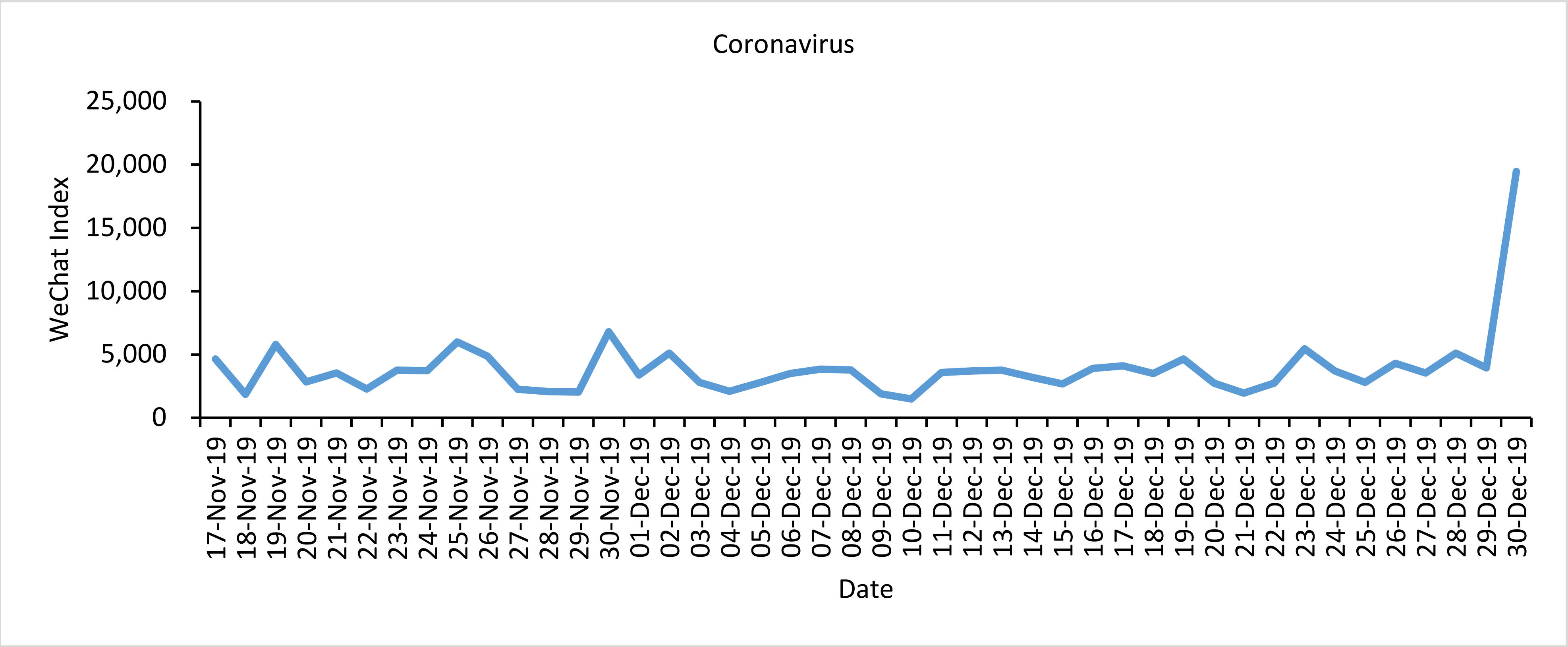
Coronavirus
WeChat Index
25,000
20,000
15,000
10,000
5,000
0
17-Nov-19
18-Nov-19
19-Nov-19
20-Nov-19
21-Nov-19
22-Nov-19
23-Nov-19
24-Nov-19
25-Nov-19
26-Nov-19
27-Nov-19
28-Nov-19
29-Nov-19
30-Nov-19
01-Dec-19
02-Dec-19
03-Dec-19
04-Dec-19
05-Dec-19
06-Dec-19
07-Dec-19
08-Dec-19
09-Dec-19
10-Dec-19
11-Dec-19
12-Dec-19
13-Dec-19
14-Dec-19
15-Dec-19
16-Dec-19
17-Dec-19
18-Dec-19
19-Dec-19
20-Dec-19
21-Dec-19
22-Dec-19
23-Dec-19
24-Dec-19
25-Dec-19
26-Dec-19
27-Dec-19
28-Dec-19
29-Dec-19
30-Dec-19
Date

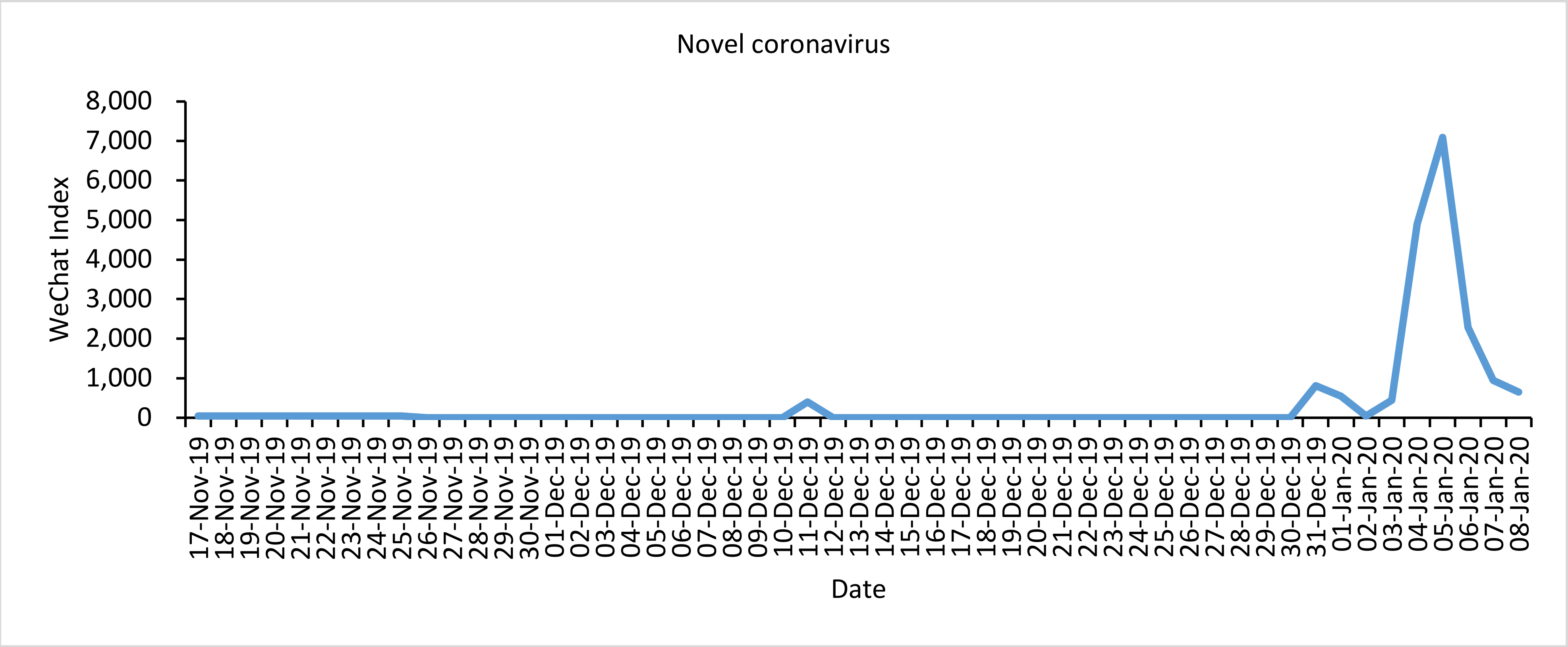

Novel coronavirus
WeChat Index
8,000
7,000
6,000
5,000
4,000
3,000
2,000
1,000
0
17-Nov-19
18-Nov-19
19-Nov-19
20-Nov-19
21-Nov-19
22-Nov-19
23-Nov-19
24-Nov-19
25-Nov-19
26-Nov-19
27-Nov-19
28-Nov-19
29-Nov-19
30-Nov-19
01-Dec-19
02-Dec-19
03-Dec-19
04-Dec-19
05-Dec-19
06-Dec-19
07-Dec-19
08-Dec-19
09-Dec-19
10-Dec-19
11-Dec-19
12-Dec-19
13-Dec-19
14-Dec-19
15-Dec-19
16-Dec-19
17-Dec-19
18-Dec-19
19-Dec-19
20-Dec-19
21-Dec-19
22-Dec-19
23-Dec-19
24-Dec-19
25-Dec-19
26-Dec-19
27-Dec-19
28-Dec-19
29-Dec-19
30-Dec-19
31-Dec-19
01-Jan-20
02-Jan-20
03-Jan-20
04-Jan-20
05-Jan-20
06-Jan-20
07-Jan-20
08-Jan-20
Date

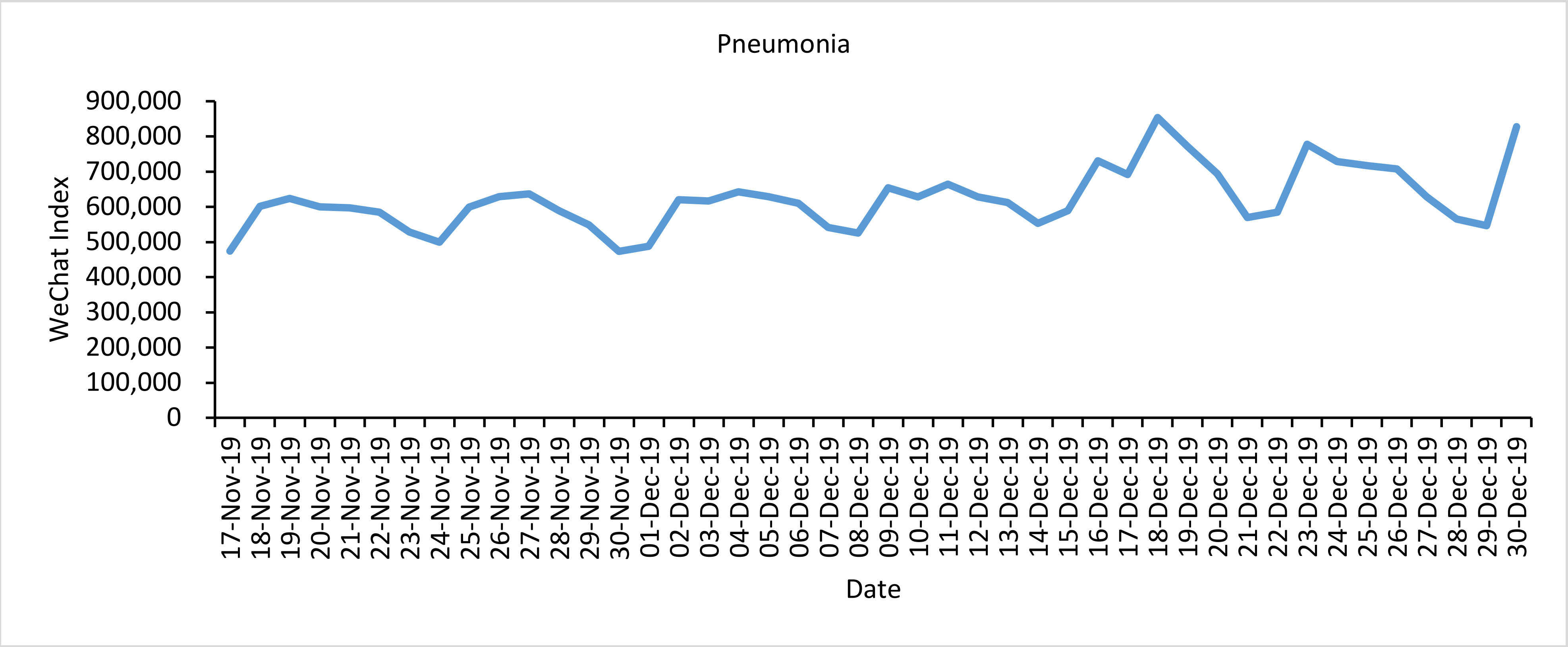
Pneumonia
WeChat Index
900,000
800,000
700,000
600,000
500,000
400,000
300,000
200,000
100,000
0
17-Nov-19
18-Nov-19
19-Nov-19
20-Nov-19
21-Nov-19
22-Nov-19
23-Nov-19
24-Nov-19
25-Nov-19
26-Nov-19
27-Nov-19
28-Nov-19
29-Nov-19
30-Nov-19
01-Dec-19
02-Dec-19
03-Dec-19
04-Dec-19
05-Dec-19
06-Dec-19
07-Dec-19
08-Dec-19
09-Dec-19
10-Dec-19
11-Dec-19
12-Dec-19
13-Dec-19
14-Dec-19
15-Dec-19
16-Dec-19
17-Dec-19
18-Dec-19
19-Dec-19
20-Dec-19
21-Dec-19
22-Dec-19
23-Dec-19
24-Dec-19
25-Dec-19
26-Dec-19
27-Dec-19
28-Dec-19
29-Dec-19
30-Dec-19
Date

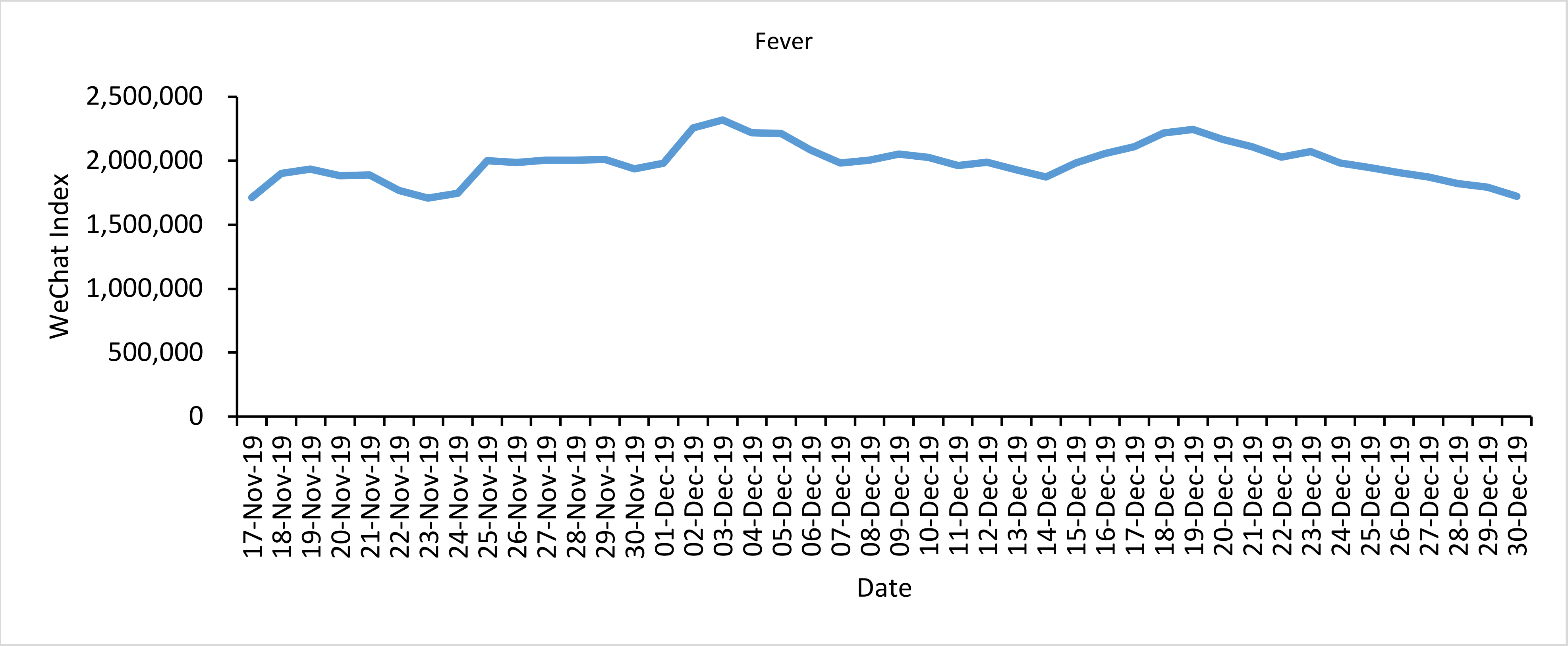

Fever
WeChat Index
2,500,000
2,000,000
1,500,000
1,000,000
500,000
0
17-Nov-19
18-Nov-19
19-Nov-19
20-Nov-19
21-Nov-19
22-Nov-19
23-Nov-19
24-Nov-19
25-Nov-19
26-Nov-19
27-Nov-19
28-Nov-19
29-Nov-19
30-Nov-19
01-Dec-19
02-Dec-19
03-Dec-19
04-Dec-19
05-Dec-19
06-Dec-19
07-Dec-19
08-Dec-19
09-Dec-19
10-Dec-19
11-Dec-19
12-Dec-19
13-Dec-19
14-Dec-19
15-Dec-19
16-Dec-19
17-Dec-19
18-Dec-19
19-Dec-19
20-Dec-19
21-Dec-19
22-Dec-19
23-Dec-19
24-Dec-19
25-Dec-19
26-Dec-19
27-Dec-19
28-Dec-19
29-Dec-19
30-Dec-19
Date

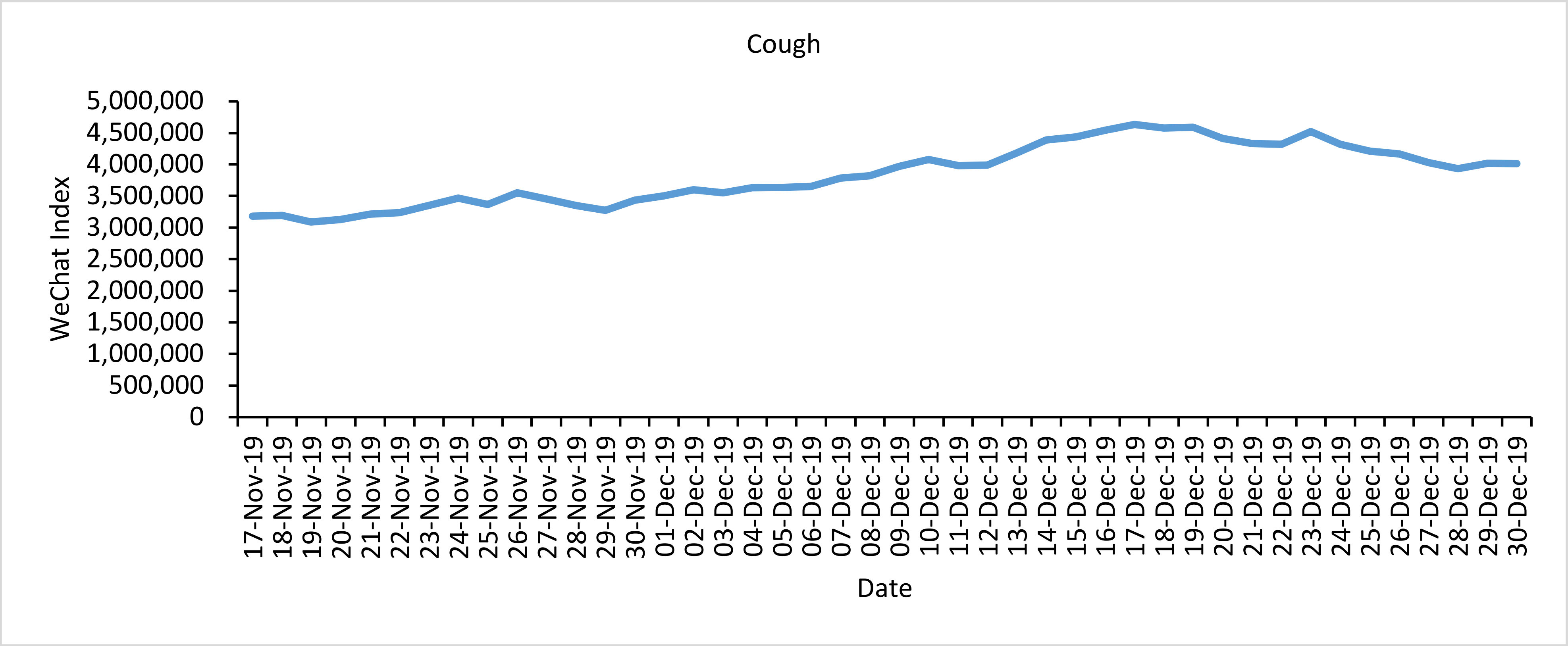
Cough
WeChat Index
5,000,000
4,500,000
4,000,000
3,500,000
3,000,000
2,500,000
2,000,000
1,500,000
1,000,000
500,000
0
17-Nov-19
18-Nov-19
19-Nov-19
20-Nov-19
21-Nov-19
22-Nov-19
23-Nov-19
24-Nov-19
25-Nov-19
26-Nov-19
27-Nov-19
28-Nov-19
29-Nov-19
30-Nov-19
01-Dec-19
02-Dec-19
03-Dec-19
04-Dec-19
05-Dec-19
06-Dec-19
07-Dec-19
08-Dec-19
09-Dec-19
10-Dec-19
11-Dec-19
12-Dec-19
13-Dec-19
14-Dec-19
15-Dec-19
16-Dec-19
17-Dec-19
18-Dec-19
19-Dec-19
20-Dec-19
21-Dec-19
22-Dec-19
23-Dec-19
24-Dec-19
25-Dec-19
26-Dec-19
27-Dec-19
28-Dec-19
29-Dec-19
30-Dec-19
Date

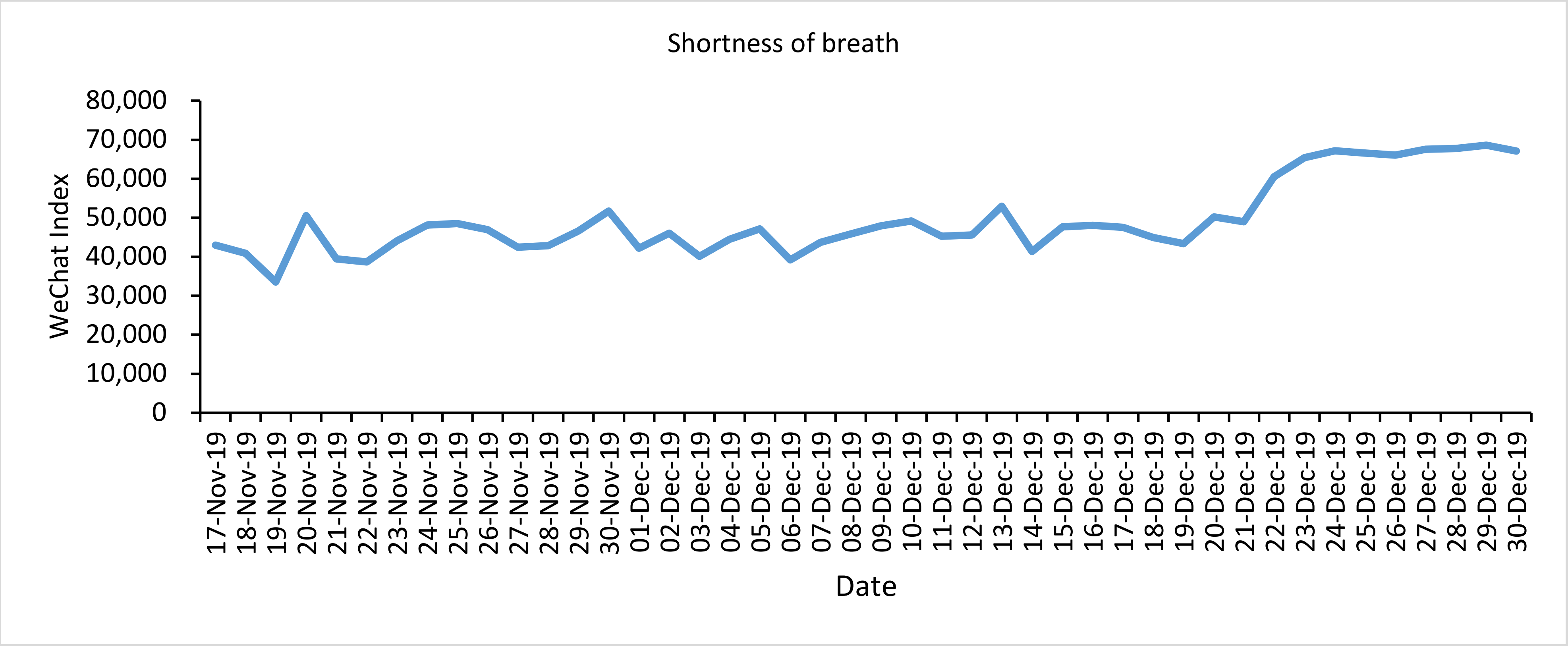
Shortness of breath
WeChat Index
80,000
70,000
60,000
50,000
40,000
30,000
20,000
10,000
0
17-Nov-19
18-Nov-19
19-Nov-19
20-Nov-19
21-Nov-19
22-Nov-19
23-Nov-19
24-Nov-19
25-Nov-19
26-Nov-19
27-Nov-19
28-Nov-19
29-Nov-19
30-Nov-19
01-Dec-19
02-Dec-19
03-Dec-19
04-Dec-19
05-Dec-19
06-Dec-19
07-Dec-19
08-Dec-19
09-Dec-19
10-Dec-19
11-Dec-19
12-Dec-19
13-Dec-19
14-Dec-19
15-Dec-19
16-Dec-19
17-Dec-19
18-Dec-19
19-Dec-19
20-Dec-19
21-Dec-19
22-Dec-19
23-Dec-19
24-Dec-19
25-Dec-19
26-Dec-19
27-Dec-19
28-Dec-19
29-Dec-19
30-Dec-19
Date

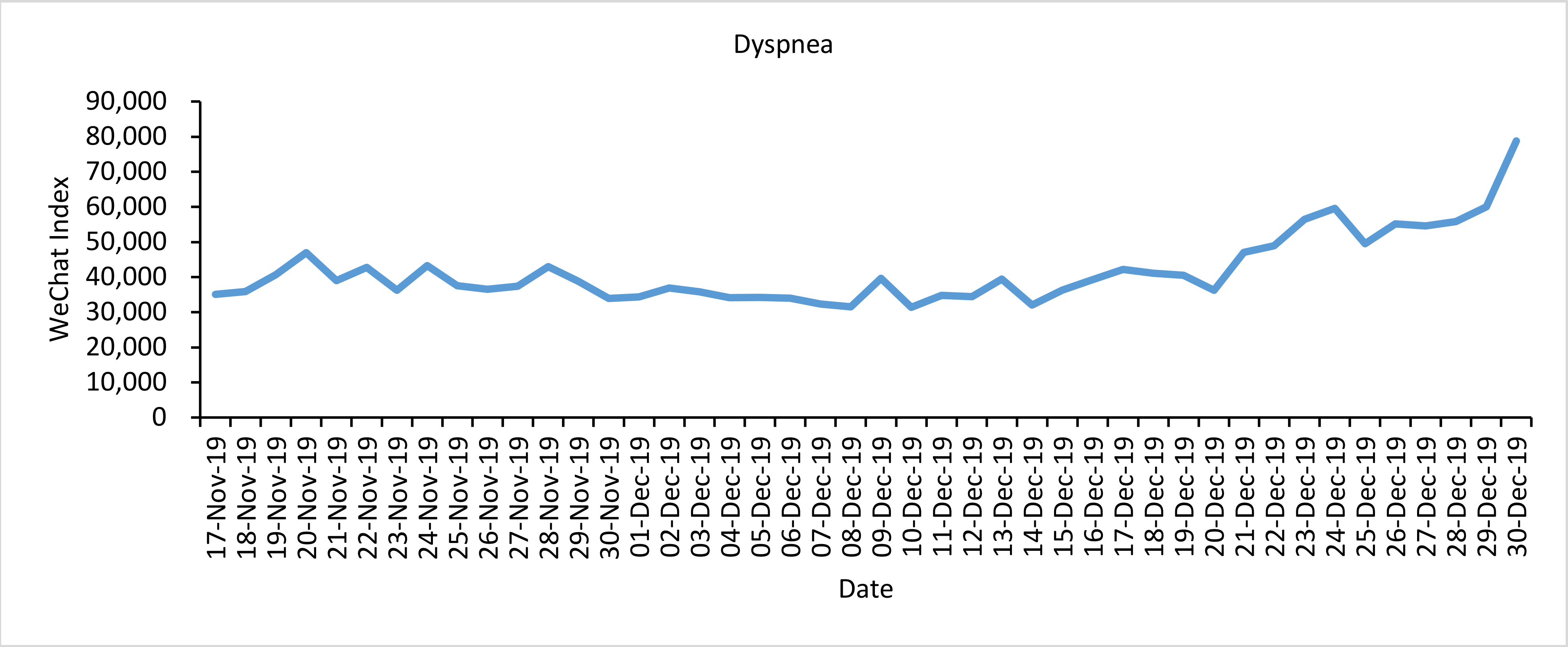

Dyspnea
WeChat Index
90,000
80,000
70,000
60,000
50,000
40,000
30,000
20,000
10,000
0
17-Nov-19
18-Nov-19
19-Nov-19
20-Nov-19
21-Nov-19
22-Nov-19
23-Nov-19
24-Nov-19
25-Nov-19
26-Nov-19
27-Nov-19
28-Nov-19
29-Nov-19
30-Nov-19
01-Dec-19
02-Dec-19
03-Dec-19
04-Dec-19
05-Dec-19
06-Dec-19
07-Dec-19
08-Dec-19
09-Dec-19
10-Dec-19
11-Dec-19
12-Dec-19
13-Dec-19
14-Dec-19
15-Dec-19
16-Dec-19
17-Dec-19
18-Dec-19
19-Dec-19
20-Dec-19
21-Dec-19
22-Dec-19
23-Dec-19
24-Dec-19
25-Dec-19
26-Dec-19
27-Dec-19
28-Dec-19
29-Dec-19
30-Dec-19
Date

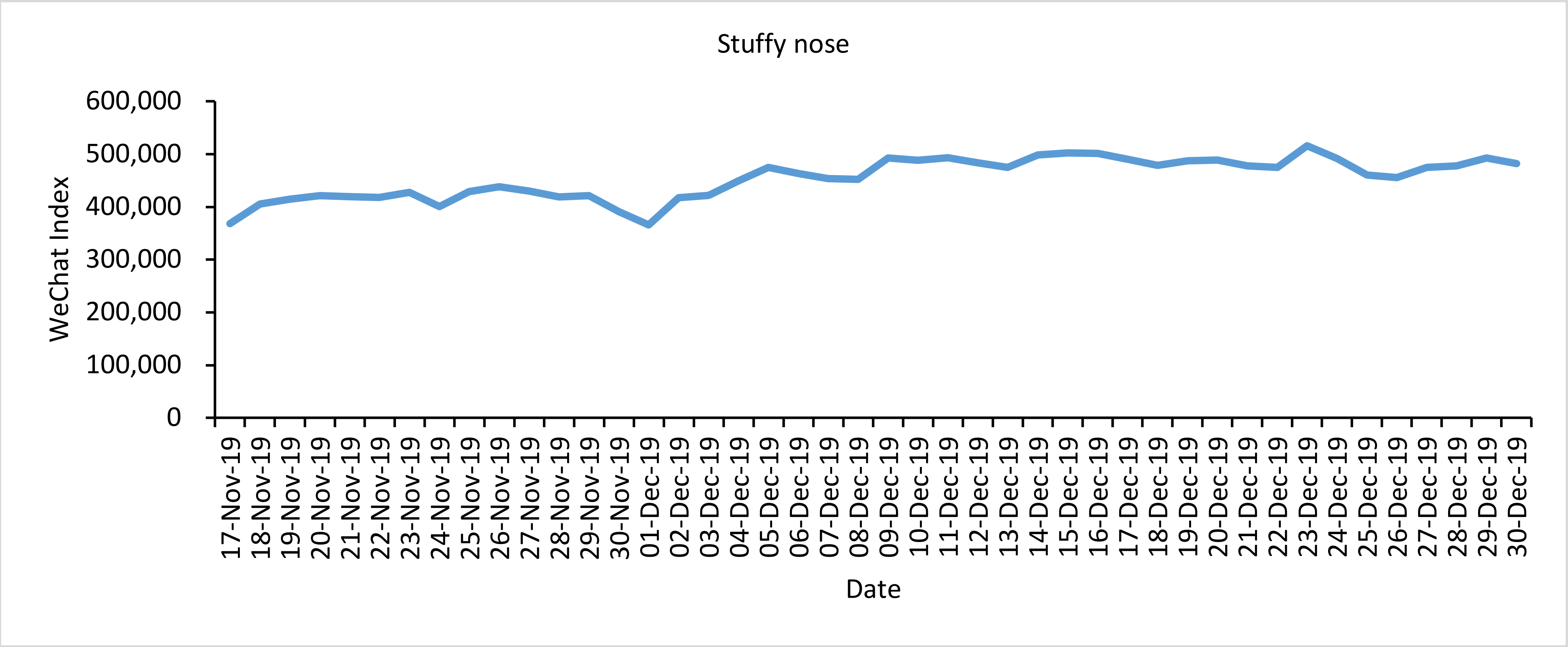
Stuffy nose
WeChat Index
600,000
500,000
400,000
300,000
200,000
100,000
0
17-Nov-19
18-Nov-19
19-Nov-19
20-Nov-19
21-Nov-19
22-Nov-19
23-Nov-19
24-Nov-19
25-Nov-19
26-Nov-19
27-Nov-19
28-Nov-19
29-Nov-19
30-Nov-19
01-Dec-19
02-Dec-19
03-Dec-19
04-Dec-19
05-Dec-19
06-Dec-19
07-Dec-19
08-Dec-19
09-Dec-19
10-Dec-19
11-Dec-19
12-Dec-19
13-Dec-19
14-Dec-19
15-Dec-19
16-Dec-19
17-Dec-19
18-Dec-19
19-Dec-19
20-Dec-19
21-Dec-19
22-Dec-19
23-Dec-19
24-Dec-19
25-Dec-19
26-Dec-19
27-Dec-19
28-Dec-19
29-Dec-19
30-Dec-19
Date

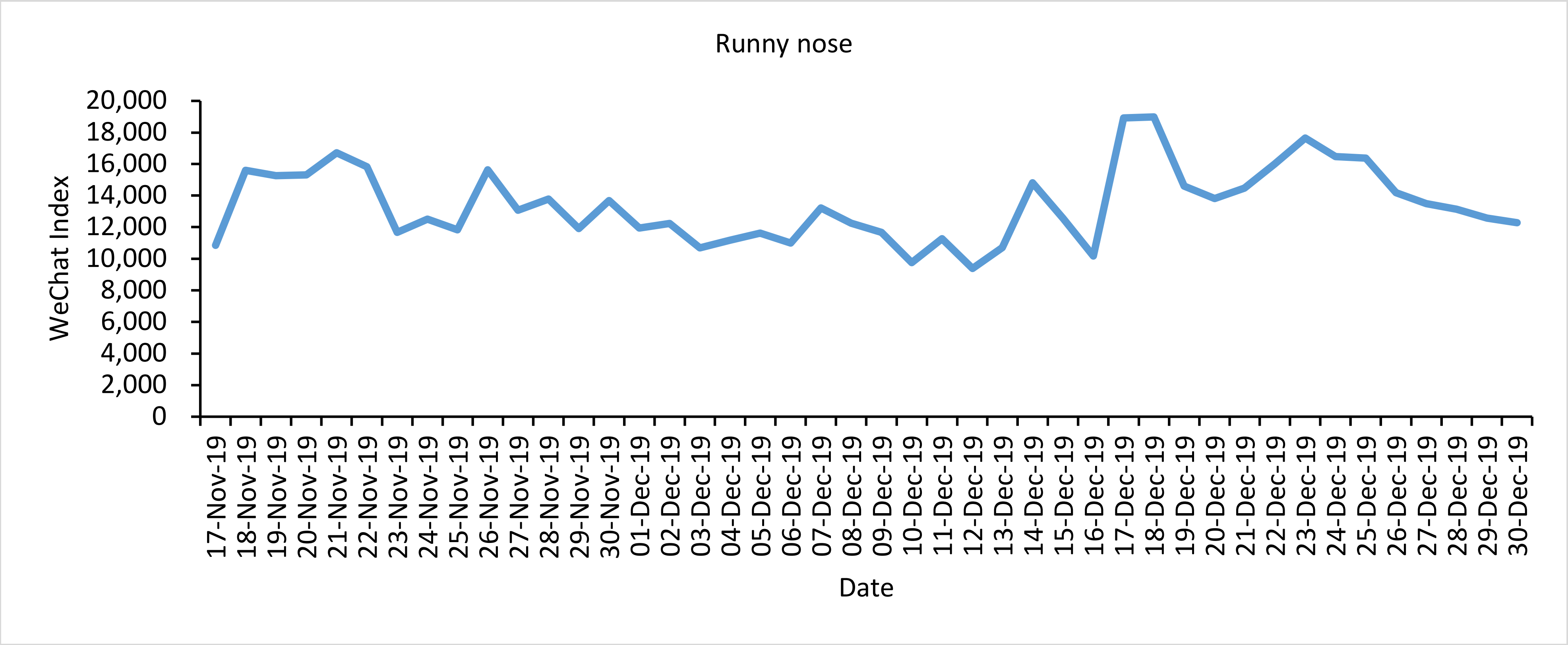
Runny nose
WeChat Index
20,000
18,000
16,000
14,000
12,000
10,000
8,000
6,000
4,000
2,000
0
17-Nov-19
18-Nov-19
19-Nov-19
20-Nov-19
21-Nov-19
22-Nov-19
23-Nov-19
24-Nov-19
25-Nov-19
26-Nov-19
27-Nov-19
28-Nov-19
29-Nov-19
30-Nov-19
01-Dec-19
02-Dec-19
03-Dec-19
04-Dec-19
05-Dec-19
06-Dec-19
07-Dec-19
08-Dec-19
09-Dec-19
10-Dec-19
11-Dec-19
12-Dec-19
13-Dec-19
14-Dec-19
15-Dec-19
16-Dec-19
17-Dec-19
18-Dec-19
19-Dec-19
20-Dec-19
21-Dec-19
22-Dec-19
23-Dec-19
24-Dec-19
25-Dec-19
26-Dec-19
27-Dec-19
28-Dec-19
29-Dec-19
30-Dec-19
Date

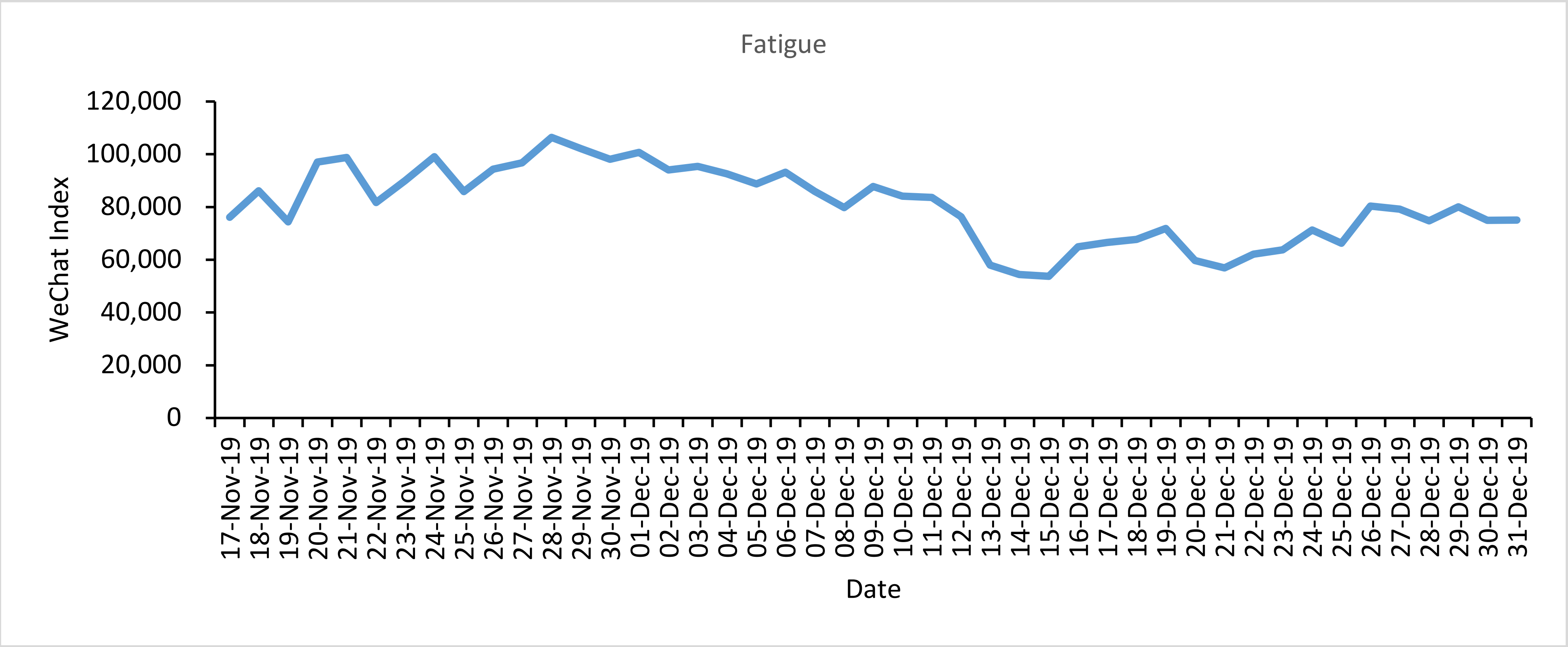

Fatigue
WeChat Index
120,000
100,000
80,000
60,000
40,000
20,000
0
17-Nov-19
18-Nov-19
19-Nov-19
20-Nov-19
21-Nov-19
22-Nov-19
23-Nov-19
24-Nov-19
25-Nov-19
26-Nov-19
27-Nov-19
28-Nov-19
29-Nov-19
30-Nov-19
01-Dec-19
02-Dec-19
03-Dec-19
04-Dec-19
05-Dec-19
06-Dec-19
07-Dec-19
08-Dec-19
09-Dec-19
10-Dec-19
11-Dec-19
12-Dec-19
13-Dec-19
14-Dec-19
15-Dec-19
16-Dec-19
17-Dec-19
18-Dec-19
19-Dec-19
20-Dec-19
21-Dec-19
22-Dec-19
23-Dec-19
24-Dec-19
25-Dec-19
26-Dec-19
27-Dec-19
28-Dec-19
29-Dec-19
30-Dec-19
31-Dec-19
Date

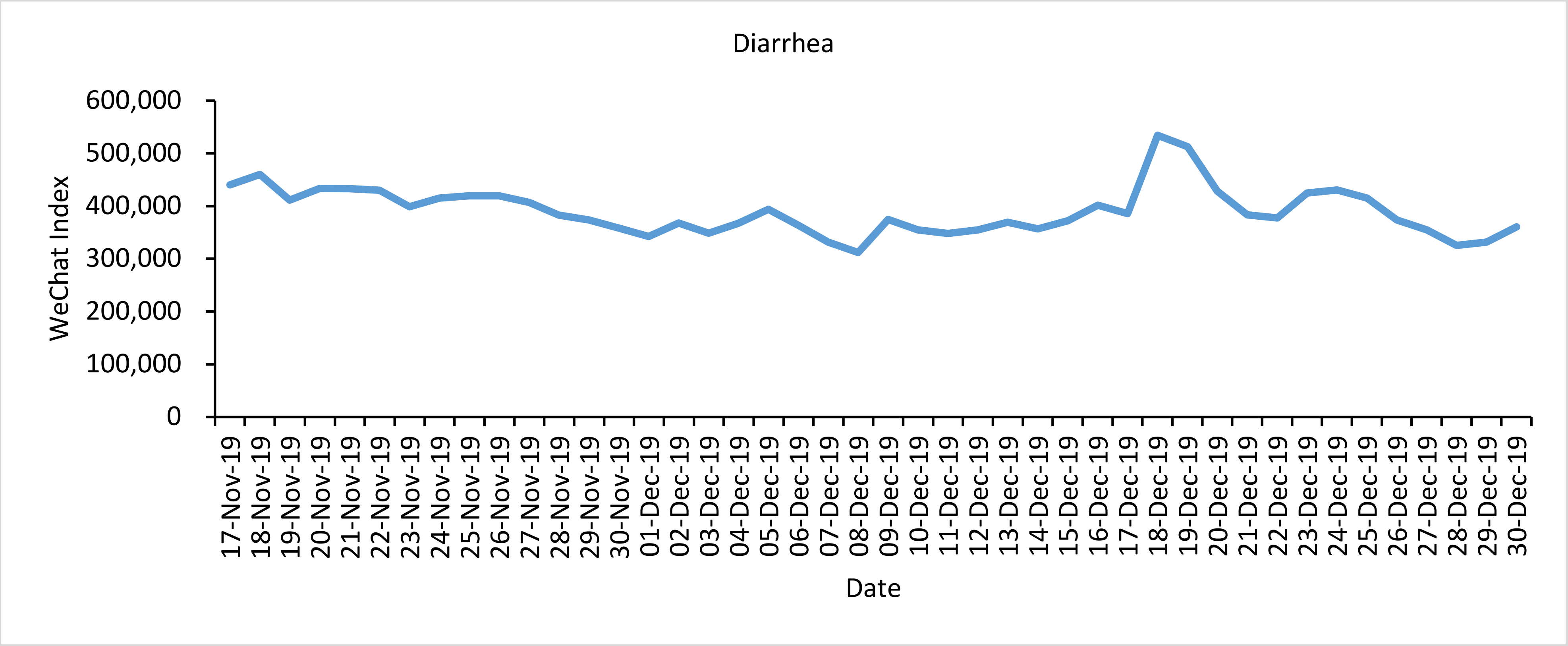
Diarrhea
WeChat Index
600,000
500,000
400,000
300,000
200,000
100,000
0
17-Nov-19
18-Nov-19
19-Nov-19
20-Nov-19
21-Nov-19
22-Nov-19
23-Nov-19
24-Nov-19
25-Nov-19
26-Nov-19
27-Nov-19
28-Nov-19
29-Nov-19
30-Nov-19
01-Dec-19
02-Dec-19
03-Dec-19
04-Dec-19
05-Dec-19
06-Dec-19
07-Dec-19
08-Dec-19
09-Dec-19
10-Dec-19
11-Dec-19
12-Dec-19
13-Dec-19
14-Dec-19
15-Dec-19
16-Dec-19
17-Dec-19
18-Dec-19
19-Dec-19
20-Dec-19
21-Dec-19
22-Dec-19
23-Dec-19
24-Dec-19
25-Dec-19
26-Dec-19
27-Dec-19
28-Dec-19
29-Dec-19
30-Dec-19
Date

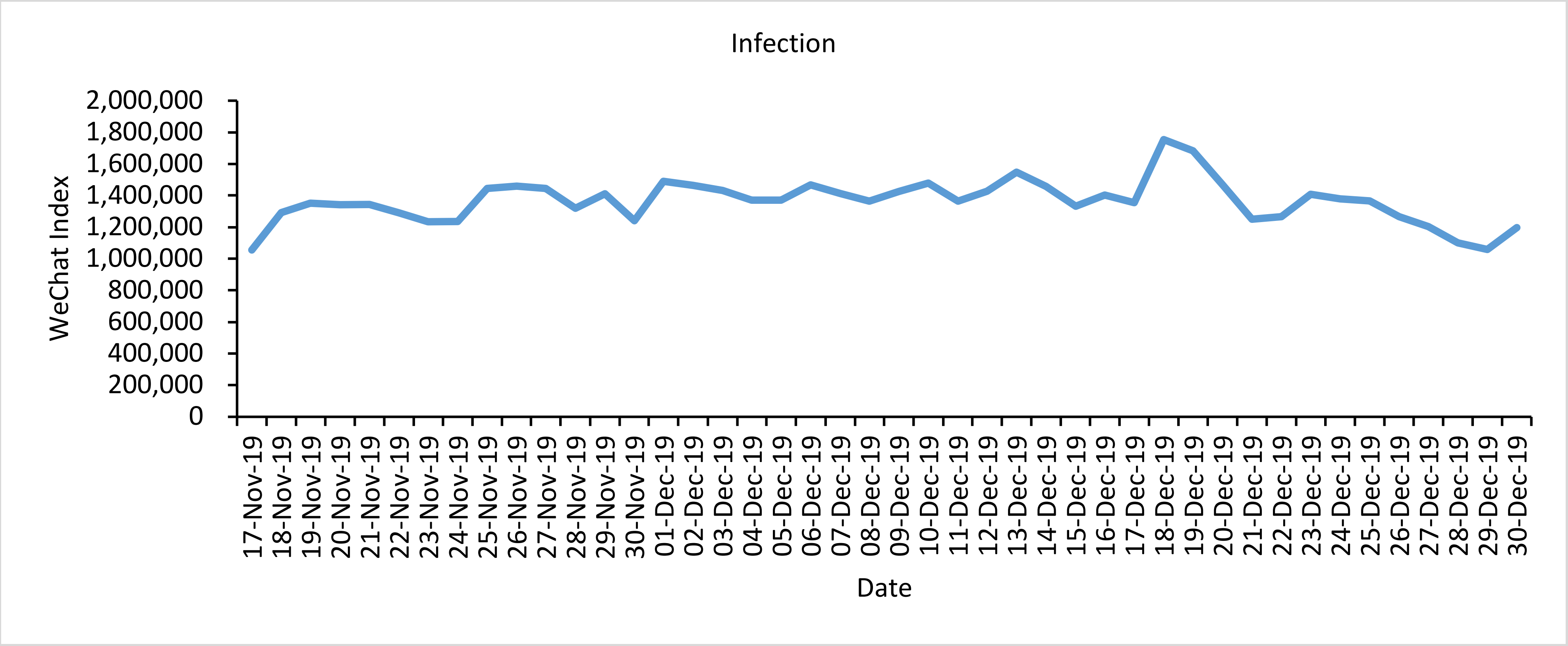
Infection
WeChat Index
2,000,000
1,800,000
1,600,000
1,400,000
1,200,000
1,000,000
800,000
600,000
400,000
200,000
0
17-Nov-19
18-Nov-19
19-Nov-19
20-Nov-19
21-Nov-19
22-Nov-19
23-Nov-19
24-Nov-19
25-Nov-19
26-Nov-19
27-Nov-19
28-Nov-19
29-Nov-19
30-Nov-19
01-Dec-19
02-Dec-19
03-Dec-19
04-Dec-19
05-Dec-19
06-Dec-19
07-Dec-19
08-Dec-19
09-Dec-19
10-Dec-19
11-Dec-19
12-Dec-19
13-Dec-19
14-Dec-19
15-Dec-19
16-Dec-19
17-Dec-19
18-Dec-19
19-Dec-19
20-Dec-19
21-Dec-19
22-Dec-19
23-Dec-19
24-Dec-19
25-Dec-19
26-Dec-19
27-Dec-19
28-Dec-19
29-Dec-19
30-Dec-19
Date
