# Supplementary material for: Using WeChat, a Chinese Social Media App, for Early Detection of the COVID-19 Outbreak in December 2019: Retrospective Study
Source: JMIR Mhealth Uhealth. 2020 Oct 5;8(10):e19589. doi: 10.2196/19589 (PMC7572119; doi:10.2196/19589)

Baidu Index for Feidian

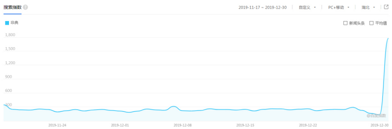

Baidu Index for SARS

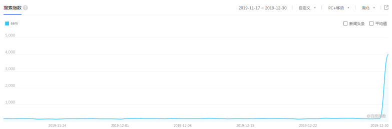

Baidu Index for coronavirus

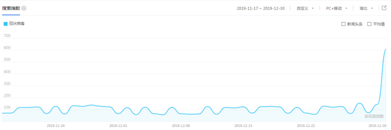

Baidu Index for novel coronavirus

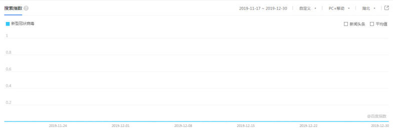

Baidu Index for pneumonia

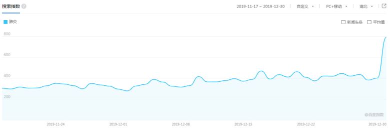

Baidu Index for fever

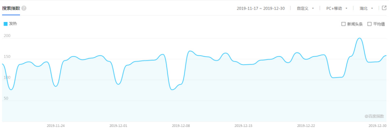

Baidu Index for cough

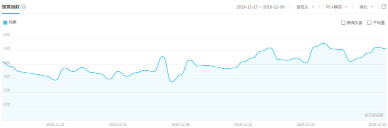

Baidu Index for shortness of breath

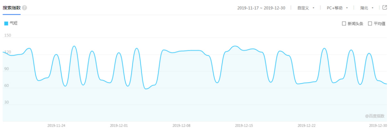

Baidu Index for dyspnea

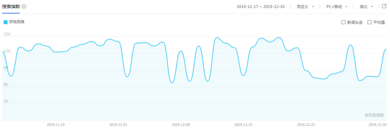

Baidu Index for stuffy nose

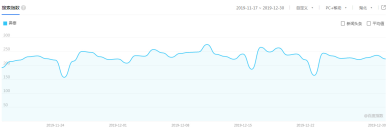

Baidu Index for runny nose

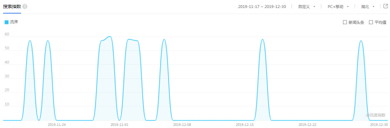

Baidu Index for fatigue

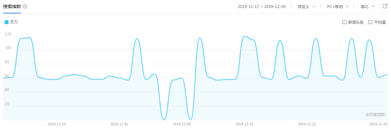

Baidu Index for diarrhea

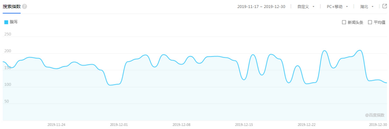

Baidu Index for infection

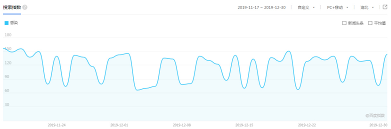

Supplement: Multimedia Appendix 4 [file mhealth_v8i10e19589_app4.pdf]
